# On-line and real time cell counting and viability determination for animal cell process monitoring by in situ microscopy

**DOI:** 10.1186/1753-6561-5-S8-P77

**Published:** 2011-11-22

**Authors:** Philipp Wiedemann, Markus Worf, Hans B Wiegemann, Florian Egner, Christian Schwiebert, Jeff Wilkesman, Jean S Guez, Juan C Quintana, Diego Assanza, Hajo Suhr

**Affiliations:** 1 At present: School of Biotechnology and Biomolecular Sciences, University of New South Wales, Sydney NSW 2052, Australia; 2 Mannheim University of Applied Sciences, Paul-Wittsack-Str.10, D-68163 Mannheim, Germany; 3 Laboratoire ProBioGEM, UPRES-EA 1024, Polytech-Lille / IUT A, Université des Sciences et Technologies de Lille, Avenue Paul Langevin, Villeneuve d’Ascq, F-59655, France; 4 InVivo BioTech Services, Neuendorfstr. 24a, D-16761 Hennigsdorf, Germany; 5 University of Carabobo, Faculty of Sciences and Technology, Chemistry Department, Valencia, 2005, Venezuela

## Background

Two of the key parameters to be monitored during cell cultivation processes are cell concentration and viability. Until today, this is very often done off-line by sterile sampling and subsequent counting using a hemocytometer or an electronic cell counter. Cell biology lacks a measurable quantity by which single cells in suspension can be non-invasively diagnosed as dead or alive. However, it would be of significance for process monitoring and in the light of initiatives like PAT if cell density as well as viability could be determined directly and on-line.

Optical measurement of cell density by *in situ* microscopy eliminates the need for sampling and allows for continuous monitoring of this key parameter; see e.g. [1,2]; Guez et al. [1] describe an in situ microscope (ISM) which does not use any moving mechanical parts within or outside the fermentation vessel. It transmits in real time images taken directly in the stirred suspension within the bioreactor. Image data is processed and evaluated to provide monitoring of cell-density and morphological parameters, e.g. cell size, by means of assessing the obtained *in situ* cell-micrographs.

Previously, we have extended *in situ* microscopy towards viability assessment of suspended cells [3,5]. Now, we present new findings on this topic and show that in cultures of suspended cells, cell-death corresponds to measurable changes in morphometric parameters as e.g. variance, contrast or entropy of the greyvalues of *in situ* cell-micrographs. As an example, here we show viability determination via greyvalue dispersion.

## Material and methods

**Figure 1:**
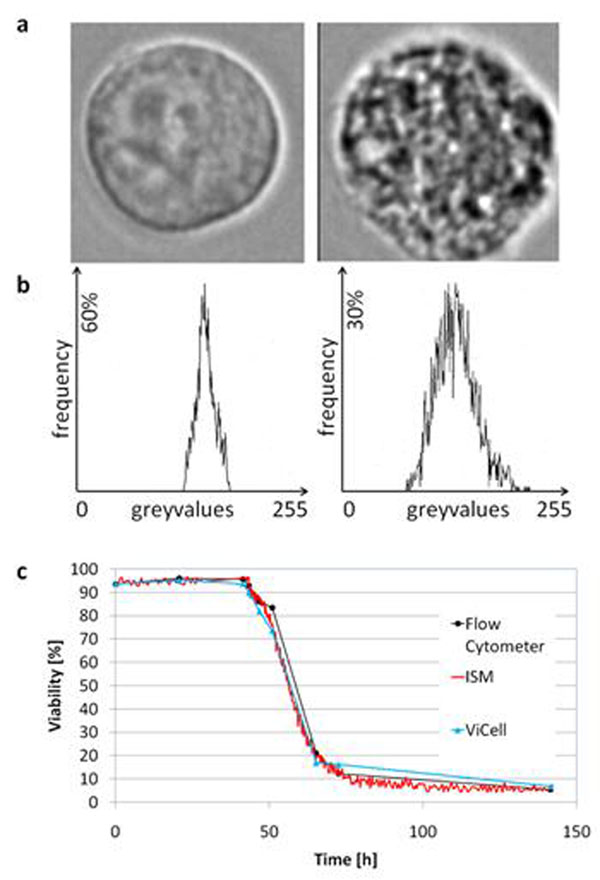
**a:** In situ micrographs of hybridoma cells. Left: Typical portrait taken in a cell culture with high viability (>90%) Right: Typical portrait taken in a cell culture with low viability (<30%). **b:** Corresponding greyvalue histograms of the respective cell 8 bit encoded portrait. 0 = white; 255 = black. **c:** Viability signals of a Jurkat batch culture as determined by ISM (as PLV-signal), ViCell (automatic counting via Trypan blue exclusion) and Flow Cytometry (AnnexinV/FITC / PI assay). The culture was insulted with EtOH (final conc. 3%) after 43 h to assess viability measurement capabilities under difficult circumstances. Similar results were obtained after addition of Etoposide (final conc. 10µM), infliction of osmotic shock or no culture insult at all.

We use a custom built high resolution ISM (HS Mannheim) with water immersion objective, 40x magnification, numerical aperture 0.75 equipped with optical fiber illumination. Data acquisition is at 0.3 – 15 frames per second, frames have 1293x1040 pixels; primary data analysis results in cell micrographs (Figure 1a). We have applied the system to bench top and larger bioreactors (see e.g. [4]) and worked mainly with Jurkat, CHO and Hybridoma cells.

For the experiment presented in Figure 1a/b, cells are cultivated in a Biostat C30 (Sartorius BBI Systems) with the ISM inserted in one of the existing probe ports. Proprietary hybridoma cells (InVivo BioTech Services) are cultured in serum free ISF-1 (InVivo BioTech Services; Biochrom AG) and monitored over the full length of the fermentation (not shown).

For the experiment presented in Figure 1c, cells are cultivated in a custom built autoclavable steel bench top reactor (HS Mannheim) with 25 mm port to accommodate the ISM and a working volume of 0.7 L. To test real time and viability determination capabilities of the system over a wide range of viabilities in a short time, cells were challenged with 3% Ethanol at 42 hours. Cell counts (not shown) and viability were determined by in situ microscopy and, as reference, by means of a ViCell cell viability analyser (Beckman Coulter) and Flow Cytometry (Partec) using Annexin V / FITC and PI staining. Jurkat cells (DSMZ ACC 282) were cultured in 90% RPMI 1640 + 10% FBS.

## Results

Figure 1a shows ISM-micrographs and corresponding greyvalue histograms during a hybridoma culture. The cell on the left side represents the typical image of cells during exponential growth at high viability. The cell on the right side represents the typical image of cells during the final stage of a culture at low viability. Cells at low viability are much less homogeneous and have a correspondingly wider dispersion of greyvalues as compared to cells at high viability. Therefore, the greyvalue-variance constitutes a random variable which is sensitive to the viability of the culture, meaning that the greyvalue-variances increase when viability drops.

In order to define - on the basis of this effect - a measurable value analogous to viability, we compute the percentage of cells with greyvalue-variance below a certain threshold. The threshold is optimized in calibration experiments applying reference methods for viability determination. We call this value “percentage of cells at low variance” (PLV). The PLV value is a viability value with respect to inhomogeneity of in situ cell-micrographs. This is analogous to viability defined as percentage of living cells with respect to a specific *ex situ* diagnosis. It has to be conceded that there is no one to one correspondence between vital cells and cells with variance below the defined threshold. Both sets – vital cells on the one side and cells with low variance on the other side– are not perfectly identical since some cells may be dead with no strong inhomogeneity, and vice versa. Despite these statistical side-effects, the PLV signal constitutes an operative measure of “inhomogeneity” and as such it also reflects the viability of the culture (see Figure 1a/b). Therefore, a high correlation can be anticipated between the PLV-signal and the viability signals from e.g. counting via Trypan blue exclusion or Flow Cytometry with AV/PI assay.

We perform online computation of PLV-values over the course of the culture to continuously monitor ISM-viability of the culture in addition to cell density (see [1]; [4]). Since data analysis and image processing happens continuously online, micrograph portraits of cells can also be assessed for other morphological characteristics as for example cell-size in real time.

Figure 1c shows results of this viability determination in the case of a Jurkat culture as an example. Similar results have been obtained with CHO and hybridoma cells. The culture was insulted by addition of 3% Ethanol to assess responsiveness of the in situ microscope. Similar correlations of viability determination by in situ microscopy and reference methods were obtained after addition of Etoposide (final conc. 10µM), infliction of osmotic shock or no culture insult at all. A very good correlation between viability determination by ISM, ViCell (i.e. Trypan Blue) and Flow Cytometry is observed (Figure 1c).

## Conclusions and perspectives

We demonstrate that our ISM is suitable for determination of suspension cell density AND viability on-line and in real time. Future work will include investigating how the fine-structure in micrographs and variance or entropy histograms corresponds to specific apoptotic stages.

For research and process applications, this work introduces non-invasive live/dead-classification of suspended mammalian cells in real time.
